# Biovalorization of whey waste as economic nutriment for mycogenic production of single cell oils with promising antibiofilm and anticancer potentiality

**DOI:** 10.1186/s13036-024-00455-y

**Published:** 2024-11-04

**Authors:** Marwa Eltarahony, Nehal El-deeb, Marwa Abu‑Serie, Hadeel El‑Shall

**Affiliations:** 1 Environmental Biotechnology Department, Genetic Engineering and Biotechnology Research Institute (GEBRI), City of Scientific Research and Technological Applications (SRTA-City), New Borg El‑Arab City, Alexandria 21934 Egypt; 2 Pharmaceutical Bioproducts Research Department, Genetic Engineering and Biotechnology Research Institute, City of Scientific Research and Technological Applications (SRTA-City), New Borg El-Arab City, Alexandria 21934 Egypt; 3 Medical Biotechnology Department, Genetic Engineering and Biotechnology Research Institute, (GEBRI), City of Scientific Research and Technological Applications (SRTA-City), New Borg El‑Arab City, Alexandria 21934 Egypt

**Keywords:** Oleaginous fungi, Secondary metabolites, Fatty acids, Antimicrobial, Biological activity, Industrial waste

## Abstract

The production of value-added bio-compounds from rejuvenated sources and their recruitment for healthcare services are paramount objectives in the agenda of white biotechnology. Hereupon, the current study focused on economic production of single cell oils (SCOs) from oleaginous fungi *Alternaria* sp. (A-OS) and *Drechslera* sp. (D-OS) using cheese whey waste stream, followed by their evaluation as antibiofilm and anticancer agents, for the first time. As a sole substrate for growth, the whey aided in lipid accumulation by 3.22 and 4.33 g/L, which representing 45.3 and 48.2% lipid content in *Drechslera* sp. (D-OS) and *Alternaria* sp. (A-OS), respectively. Meanwhile, a higher unsaturation degree was detected in A-OS by 62.18% comparing to 53.15% of D-OS, with advantageous presence of omega-6 poly unsaturated fatty acid by 22.67% and 15.04% for A-OS and D-OD, respectively, as revealed by GC-MS and FTIR characterization analysis. Interestingly, an eminent and significant (*P* ≤ 0.05) antibiofilm potency was observed in a dose-dependent modality upon employing both SCOs as antibiofilm agents. Whereas, 100 µg/mL of A-OS recorded superior inhibition of *P. aeruginosa*, *S. aureus* and *C. albicans* biofilms development by 84.10 ± 0.445, 90.37 ± 0.065 and 94.96 ± 0.21%, respectively. Whereas, D-OS (100 µg/mL) thwarted the biofilms of *P. aeruginosa*, *S. aureus* and *C. albicans* by 47.41 ± 2.83, 62.63 ± 5.82 and 78.67 ± 0.23%, correspondingly. Besides, the metabolic performance of cells within biofilm matrix, protein, carbohydrate contents and hydrophobicity of examined biofilms were also curtailed in a significant correlation with biofilm biomass (*r* ≥ 0.9). Further, as anticancer agents, D-OS recorded higher potency against A549 and CaCo-2 cell lines with IC50 values of 2.55 and 3.425% and SI values of 10.1 and 7.5, respectively. However, A-OS recorded 8.275% and 2.88 for IC50 and SI of Caco-2 cells, respectively. Additionally, A-OS activated caspase 3 by 64.23 ± 1.18% and 53.77 ± 0.995% more than D-OS (52.09 ± 0.222% and 49.72 ± 0.952%) in A549 and Caco-2 cells, respectively. Furthermore, the enzymes, which associated with cancer invasion, metastasis, and angiogenesis (i.e., MMP2 and MMP9) were strongly inhibited by A-OS with 18.58% and 8.295%, respectively as IC50 values; while D-OS results recorded 23.61% and 13.16%, respectively, which could be ascribed to the higher ω-6/ω-3 contents of A-OS. The promising results of the current study opens up the vision to employ SCOs as anti-infective nutraceuticals and in complementary/alternative therapy and prophylactic programs as well.

## Introduction

The industrial revolution is the fundamental engine behind numerous advanced technologies, which introduces perceptible leaps in the provision of multiple services, society and economics. Nonetheless, the production of huge amounts of contaminants that released continuously in our biosphere considers being the main obstacle of such technological progress. The pollution of natural resources (i.e., water, soil, air) with anthropogenic waste products undoubtedly harms human health and its surrounding environment, in particular with overpopulation and uncontrolled urbanization. Notably, cancer and biofilm causing illness are the most common diseases caused by environmental pollution and one of the most representative etiological agents for morbidity and mortality as revealed by world health organization (WHO) and National Institutes of Health (NIH) [1–3].

However, the remedy of biofilm infections and firm tumors confronts a similar complication. Wherein, the notable conventional medications face certain difficulties in reaching and damaging the inner cells of cancer and biofilm-embedded bacteria owing to the heterogeneities of local micro-environment [4, 5]. Additionally, the alteration in immune reactions and cytotoxicity of applied chemotherapy against healthy cells were also documented in both diseases. The microbiota dysbiosis, neuromuscular blockade, nephrotoxicity and ototoxicity are the frequent side effects of antimicrobial agents. However, the lower counts of neutrophil, platelet and erythrocyte are the common complications of cancer therapeutic [4–6].

Accordingly, the necessity for design new, biosafe and effective antibiofilm/ anticancer drugs, for coping with such upsetting difficulties, is urgent. In the recent era, the biologically-based technology (i.e., metabolomics and biotechnology) gained colossal attention from scientists and technologists by the dint of its safety, biocompatibility, eco-friendliness and sustainability. Intriguingly, such biologically-based products are derived from plants, animals and microorganisms, which provide prototypes for several pharmacologically active compounds in treating cancer and biofilm-based infections [7]. Single cell oils (SCOs) or Fatty acids (FAs) are categorized among distinct and potential bioactive compounds, which pervasive in nature and have an indispensable role in biological, nutritional and clinical viewpoints [8]. It is worth recalling that consumption of certain FAs has been powerfully associated with several health profits, especially when interchanging saturated fatty acids (SFAs) with mono-un saturated fatty acids (MUFAs) or poly unsaturated fatty acids (PUFAs). They play a crucial role in reducing cholesterol, which in turn reduce the danger of cardiovascular disease and have been reported to reduce the risk of inflammatory conditions such as arthritis, Crohn’s disease and asthma. Besides, MUFAs and PUFAs are recognized as apoptosis-dependent anticancer dietary components, which causing selective cytotoxicity towards cancer cells with little or no toxicity on normal cells [9, 10]. The carcinogenic process is believed to involve MMP-2 and MMP-9 in order to angiogenesis and metastasis occurring in various cancer cell lines. Previous studies demonstrated that matrix metalloproteinase (MMP) activities can be inhibited by PUFAs [11–13]. Unfortunately, human bodies cannot synthesis PUFAs and therefore, it is essential to be obtained them from only dietary sources in our life style and this essentiality is also spirited for cancer cells [14].

Collectively, these overall features seem being advantageous upon employing industrial waste products as raw materials for the manufacturing process; taking into account the green production in an economic way. Food industries, in particular dairy industry, are continuously developed in an incredible dynamic process of transformation to meet market requirements. Due to the economic and nutritional significance of dairy products, their industry deemed a substantial societal asset globally, generating hereby large quantities of whey as co-product or feedstock [15]. Noteworthy mentioning that whey is organic effluent rich in easy fermentable sugar (i.e., lactose), fats, proteins, non-protein nitrogenous compounds, lactic acid, vitamins and minerals with biochemical oxygen demand (BOD) and chemical oxygen demand (COD) oscillated from 40 to 60 and 50 to 80 g/L, respectively. Thus, its arbitrary discharge in water bodies, without proper management and sustainable practices, represents a real environmental risk [16, 17]. Let alone the economic losses due to squander of nutrients and energy. Therefore, for maintaining the nutritional value of whey and simultaneously alleviating its detrimental environmental peril, various studies utilized it as cost-effective and sustainable substrate for resource recovery, microbial growth and yielding advanced valuable biotechnological products [18, 19].

Based on the previous background, our study focused on the economic production of mycogenic SCOs, under the umbrella of reduced operational costs, using cheese whey as sole and chief nutritional media. The extracted SCOs were characterized and subsequently evaluated as antibiofilm and anticancer agent. Hitherto, no study till our knowledge, scrutinized the antibiofilm and anticancer potentiality of SCOs generated by both fungal strains under study.

## Materials and methods

### Fungal stains, growth conditions, extraction, transesterification and characterization of lipids samples

The two fugal strains of this study *Alternaria* sp. and *Drechslera* sp. were isolated, examined as oleaginous filamentous fungi and identified molecularly with accession numbers of MH348917.1 and MG582185.1, respectively [20, 21]. Strains were maintained on potato dextrose agar slants (PDA) at 4 °C and refreshed every 3–4 months. Cheese whey, as waste stream, was procured from dairy processing factories in New Borg El-Arab city and utilized as main cultivation media; replacing entirely, by such way, the microbiological media for fungal propagation and lipid accumulation. By using 1.0 M (HCl or NaOH), its pH was adjusted to 6 followed by sterilization. In 250 ml flasks contained about 70 ml of whey, the fungal lawns were inoculated and incubated at 28^o^C in an orbital shaking 150 rpm for 4 days, in triplicate. Thereafter, dry biomass, lipid yield and content were determined as described in details by El-shall et al. [9]. Extraction and transesterification of lipids were performed as briefly described in [22]. The transesterified lipids from both fungal strains were characterized by gas chromatography–mass spectrometry (GC-MS) and Fourier Transform Infrared Spectroscopy (FTIR). Initially, the samples were analyzed by Agilent 6890 Gas Chromatograph equipped with a straight deactivated 2 mm direct injector liner and a 15 m Alltech EC-5 column (250 µ I.D., 0.25 µ film thickness), to detect and quantify saturated and unsaturated contents of fatty acid. The operating conditions were held at 250 °C for inlet temperature, 280 °C for detector temperature and 35 °C initial oven temperature, which was held for 2 min then elevated to 300 °C for 23 min. The injection volume was 2 mL, with a split ratio of 10:1. Helium served as a carrier gas at a constant flow rate of 1 ml/min. The FAs profile was identified via comparison of its chromatographic peaks and retention times with those of WILEY 09 and NIST 11 mass spectral database. Notably, each individual peak was quantified by means of standards and their corresponding calibration curves. Regarding FTIR, KBr was used as a matrix in a disc method through a scanning spectrum ranged from 3500 to 500 cm^−1^ by Shimadzu FTIR-8400 S, Japan spectrophotometer at a resolution of 4 cm^−1^ [23].

### Antibiofilm activity of lipid samples

The microtiter plate assay was applied to determine the antibiofilm activity of the SCOs qualitatively. Herein, *Candida albicans* (ATCC 10231), *Staphylococcus aureus* (ATCC 25923) and *Pseudomonas aeruginosa* (ATCC 27853) were used as representative strains for yeast forming biofilm, Gram-positive and Gram-negative bacteria, respectively. In brief, about 10 µL (1 × 10^5^ CFU/mL) of overnight microbial culture was dispensed in U-bottom microtiter plate contained sterile Trypticase Soy Broth (TSB) supplemented with 1% w/v glucose (TSBG). Meanwhile, different concentrations of both SCOs (1–100 µg/mL) were pipetted in each inoculated well. Remarkably, wells contained TSBG without microbial inoculum containing 0.1% DMSO and inoculated wells lacking SCOs -treatments were run simultaneously as negative controls and positive controls, respectively. The plates were statically incubated for 24 h at 37 °C to empower the microbial propagation and biofilm maturation. Following incubation, the spent medium containing free-floating cells were decanted gently from each well. Then, 200 µl of sterile saline (0.9% NaCl) was used thrice to wash each well. Subsequently, the adherent cells were stained using Hucker’s crystal violet (0.1%, w/v) for 20 min at 37 °C. The excess dye was removed after incubation and the stained biofilms were washed off gently with deionized distilled water. To elute the attached cells, 200 µL of 95% ethanol was pipetted in each well and the absorbance was measured at 595 nm spectrophotometrically by microtiter ELISA reader (Tecan Infinite M200, Switzerland) to quantify biofilms [24]. The percent of biofilm suppression was calculated as described in the following formula:

1 $$\text{Biofilm inhibition}\,\%=\left(\text{A}-\text{A}_{\mathrm o}\right)/\text{A}\ast 100$$

Where A and A_0_ pointed out to the absorbance of the positive control and the treated wells, respectively.

#### The effect of SCOs samples on biofilm metabolic activity

The viability of biofilm cells and their respiratory activity was quantified using (MTT) assay. The assay based on the capability of surviving and metabolically active cells to reduce the yellow tetrazolium salt a (3-[4,5-dimethyl-2-thiazolyl]-2, 5-diphenyl-2 H-tetrazolium-bromide) to a purple formazan. The setting up of the experiment was previously described. Once the biofilm formed after 24 h incubation and washed, about 200 µL of 0.25 mg/mL MTT solution was added and gently pipetted with the content of each well. The 96-well tissue culture plate was incubated under dark and static condition for 2–3 h at 37 °C. The solution was withdrawn and replaced with 2% of dimethyl sulphoxide (DMSO) for solubilizing the insoluble formazan crystals. The absorbance was recorded at 570 nm using microtiter ELISA reader [23]. The higher absorbance reading reveals higher number of surviving cells in the biofilm. The effect of both oils on biofilm cells was calculated as the previous equation (Eq. 1).

#### The effect of SCOs samples on carbohydrate and protein contents of the biofilms

The biochemical components of the isolated biofilm in terms of total carbohydrate/ePS and total protein were detected before and after SCOs treatment. The phenol–sulfuric acid protocol was employed to quantify the carbohydrate content using glucose as a standard; while Bradford assay was utilized for determination protein concentration and BSA used as a standard [25].

#### The effect of SCOs samples on biofilm hydrophobicity

The hydrophobicity of untreated or control and SCOs-treated biofilm cells were measured through microbial adhesion to hydrocarbon (MATH) assay as described by [26]. Via such biphasic hydrocarbon/aqueous assay, the cell surface hydrophobicity (CSH) was expressed based on the change in optical density of the aqueous phase relative to the control. Initially, the biofilms were allowed to grow followed by decanting the planktonic cells, washing and suspension in phosphate buffer saline (PBS) (200 µL). Through scraping by a pipette tip, the biofilms became detached and resuspended in 3 mL of PBS buffer, then homogenously disruption by vortexing for 3 min; 0.4 mL of xylene (hydrocarbon) was added to 2 mL of biofilm suspensions and vortexed vigorously for 3 min. The overall suspensions were allowed to stand at room temperature for 15 min till the separation of hydrocarbon/ aqueous phase. Ultimately, the absorbance of biofilm cells that remained in aqueous phase (i.e., hydrophobicity %) was determined spectrophotometrically at 600 nm by the following equation (Eq. 2).

2 $$\begin{aligned} &\text{Hydrophobicity inhibition}\,\%\\ & =1-\left(\text{OD600 nm positive control}\right)\\ &\quad -\left(\text{OD600 nm treated}\right)]/\\ &\qquad (\text{OD600 nm positive control})\times 100 \end{aligned}$$

The cells described as strongly hydrophobic, moderately hydrophobic and hydrophilic when the obtained values recorded > 50%, 20–50% and < 20%, respectively [23, 27].

### Anticancer activity of lipid samples

#### Cell lines

Wi-38 cell line (Human normal fibroblast lung cells), CaCo-2 cell line (Human Colorectal adenocarcinoma, epithelial cells) and A549 cell line (Human Lung adenocarcinoma, epithelial cells) cell lines were purchased from ATCC.

#### Safety assay and anticancer activity

An In-vitro viability test was used to determine the safety patterns and the anticancer effects of the tested SCOs samples on non-cancerous (Wi-38) and cancerous cell lines (A549 and Caco-2) using 3-(4,5-dimethylthiazol-2-yl)-5-(3-carboxymethoxyphenyl)-2-(4-sulfophenyl)-2 H-tetrazolium (MTS) (Promega) assay according to the manual instructions. Briefly, 100 µL of (6 × 10^4^ cells/mL) of the each overnight cell line culture were inoculated into 96 well plates. The inoculated plates were incubated overnight till semiconfluency. After incubation, 100 µL of oil samples at different concentrations (prepared in DMEM media) were added to the plates and the plates were incubated for further 2 days. The cellular viability was determined by quantifying the solubilized formazan in DMSO at 570 nm. The inhibition concentration at 50% (IC50) was quantified from the cytotoxicity % curve using GraphPad prism 9.

#### Selectivity index of oil samples

Cancer cell selectivity index of the examined SCOs-treated samples was calculated according to the method explained by [28] with a minor modification; SI = IC50nc/IC50cc, where IC50nc refers to the value of IC50 of the oil samples effects on normal cells, while IC50cc refers to the IC50 of the oil samples effects on cancer cell line.

#### Determination of caspase 3 activation

In brief, the untreated and treated cell lines were lysed in the supplemented lysis buffer and centrifuged. Supernatants were incubated with the kit reaction buffer and substrate at 37 °C, as illustrated in caspase-3 assay colorimetric kit (Abcam, US). After 2 h, the absorbance of all samples were assessed at 405 nm using microplate reader.

#### Determination of MMP2 and MMP9 inhibition

Following kit instructions (Abcam, US), serial concentrations of both SCOs were incubated with assay buffer and enzyme (MMP2 or MMP9) for 45 min at 37 °C. After adding substrate, absorbances were measured at 412 nm.

### Statistical analysis

In the present study, all experiments were performed trice and the results were averaged and represented as means ± SEM (Standard Error of Mean). All results were analyzed by one way ANOVA followed by Tukeys test. The statistical significance of collected data was accounted when *P*- value was ≤ 0.05, as per Graphpad Prism 5.03 software (Graph Pad Software Inc., La Jolla, CA, USA) [23].

## Results and discussion

### Economic production and characterization of fungal SCOs 

The microbial lipids or SCOs, are categorized among the most promising natural feedstock for biofuel and nutraceuticals production. Remarkably, the proximity of their structure with fish oil or even vegetable oils had gained a momentum, especially those derived from fungi. That could be attributed to their large quantity of biomass with high lipids yield in a short growth cycle and facile biomass collection. Remarkably, the versatility in fungal growth conditions facilitated their cultivation on low-cost culture medium based on different wastes, which were continuously accumulated in the environment causing severe pollution. Such dual tasks, of investing the environmental contaminants in biotechnological products, consider being the fundamental pillar of sustainable green techno-economic productivity [9]. Several species of filamentous fungi were recorded in bioconversion of food processing wastes into value-added by-products such as *Aspergillus*,* Rhizopus and Trichosporon* [17]. Interestingly, *Drechslera* sp. and *Alternaria* sp. demonstrated significant potential for lipid production, especially under using of agro-industrial wastes as substrates [20, 21]. Hereupon, the current study focused on mycological production of SCOs from both fungal strains in an economic process via utilizing by-product of the cheese manufacturing industries (i.e., whey), as a sole nutrition source. Generally, it was recorded as highly nutritive waste stream full of proteins, vitamins, sugars, minerals, and other growth factors [29] and also reported previously as supporting material in fungal lipid production [30, 31]. Herein, the whey was utilized as a cultivation media without any addition of other nutrient sources. It facilitated the lipid accumulation yield in both filamentous fungi by 3.22 and 4.33 g/L, which representing 45.3 and 48.2% lipid content in *Drechslera* sp. and *Alternaria* sp., respectively. In comparison, the lipid content of *Drechslera* sp. and *Alternaria* sp. reached to 33.18 and 29%, with lipid yield evaluated by 3.65 and 5.6 g/L, respectively, upon replacing carbon source in their optimized media with agricultural wastes (i.e., orange peel and molasses). However, on optimized microbiological media (i.e., Czapek-Dox’s medium), their lipid contents were assessed by 40.75 and 50.3% for *Drechslera* sp. and *Alternaria* sp., respectively, reflecting the higher potentiality of whey stream as the main cultivation and production media in the lieu of carbon or nitrogen source [20, 21].

**Fig. 1:**
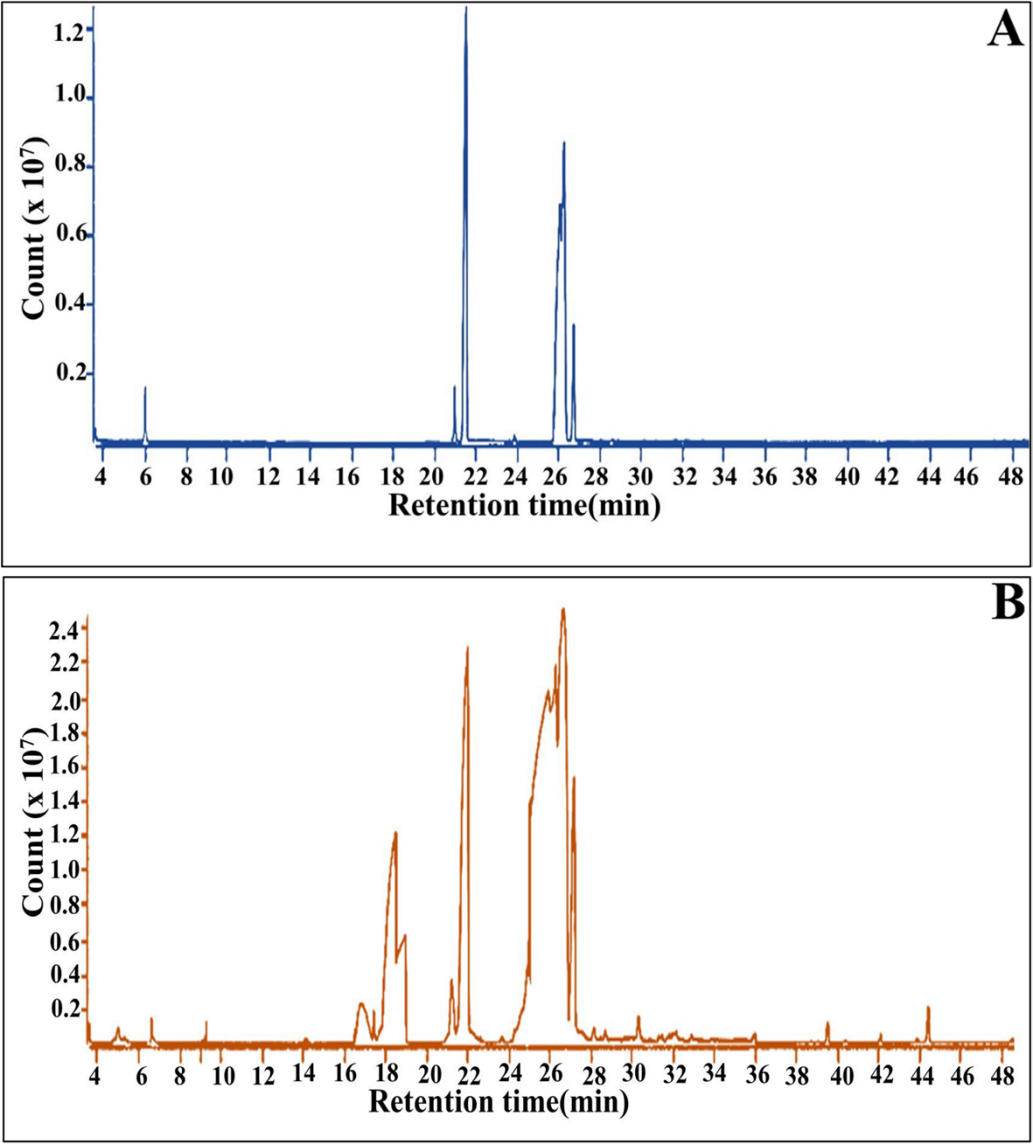
GC-Ms analysis of fatty acid produced by (**A**) *Alternaria* sp. **B** *Drechslera* sp. cultivated on whey

The characterstic features of SCOs from both oleaginous fungi were determined initially by identifying their components through investigating the fatty acid methyl esters profiles after acidic transesterification as shown in (Fig. 1) and (Table 1), which manifested the qualitative and quantitative differences in both examined profiles. Notably, the results of gas chromatography–mass spectrometry (GC-MS) showed common features among both profiles. Wherein, unsaturated fatty acids (USFAs) represented the dominate constituent in the profiles of both fungi by 62.18 and 53.15% for *Alternaria* sp. and *Drechslera* sp. respectively. Meanwhile, Palmitic acid-C16 (SFAs) was the most prevalent FA by 29.0 and 28.9% for *Alternaria* sp. and *Drechslera* sp. respectively, followed by Oleic acid -C18 (MUSFAs), which was evaluated by 24.7 and 28.8%, % for *Alternaria* sp. and *Drechslera* sp., correspondingly. On the other hand, the omega-6 (ɷ-6) poly unsaturated fatty acid (PUFAs) (i.e., Linolenic acid and *γ*-linolenic (C18) was the third major constituent in both profiles with the values of 26.89% and 15.72% for *Alternaria* sp. and *Drechslera* sp. respectively. Interestingly, an obvious percent of PUFAs (30.02%) was detected for *Alternaria* sp. compared to 17.93% for *Drechslera* sp. In accordance with our result, Lauryn et al. [32] demonstrated that cheese whey was ideal choice for *Mucor circinelloides* in SCOs production with GC-MS-profile contained predominantly oleic acid (41%), palmitic acid (23%), linoleic acid (11%), and γ-linolenic acid (9%).

**Table 1:** Showing fatty acids (FAs) patterns of both SCOs extracted from *Alternaria *sp.and* Drechslera *sp.after their cultivation on whey media

Fatty acids	Retention time	SCOs of Alternaria sp. A-OS	SCOs of Drechslera sp. D-OS
Caprylic acid (C8)	7.007	0.88	0.23
Myristoleic acid (C14)	16.838	0.52	0.43
Myristic acid (C14)	17.453	0.38	0.55
Palmitoleic acid (C16)	21.233	4.49	4.36
Palmitic (C16)	22.009	29.01	28.89
Heptadecenoic	24.299	0.34	0.19
Gama-linolenic (C18)	26.144	4.22	0.68
Linolenic (C18)	26.309	22.67	15.04
Oleic (C18)	26.144	24.78	28.86
Elaidic (C18)	26.350	1.71	0.21
Stearic (C18)	26.555	3.51	13.31
Cis-5,8,11,14,17-Eicosapentaenoic (C20)	31.315	0.63	0.40
Cis-8,11,14-Eicosatrienoic (C20)	31.798	0.65	0.15
Cis-11,14-Eicosadienoic (C20)	32.166	0.61	0.18
Cis-11-Eicosenoic (C20)	32.304	0	0.17
Cis-11,14,17-Eicosatrienoic (C20)	32.407	0.6	0.14
Arachidic (C20)	33.127	0.48	0.27
Heneicosanoic (C21)	36.513	0.43	0.11
Cis-4,7,10,13,16,19-Docosahexaenoic (C22)	37.778	0.64	1.21
Cis-13,16-Docosadienoic (C22)	38.745	0	0.14
Erucic (C22)	38.757	0	0.79
Behenoic (C22)	39.521	0.94	0.99
Tricosanoic (C23)	42.082	0.62	0.54
Nervonic (C24)	43.905	0.67	0.40
Lignoceric (C24)	44.429	1.23	1.77
**Fatty acid type**	**Total % of FAs**		
**SFAs**	37.82		46.85
**USFAs**	62.18		53.15
**MUFAs**	32.17		35.22
**PUFAs**	30.02		17.93
**ɷ-3**	1.87		1.75
**ɷ-6**	28.15		16.18

**Fig. 2:**
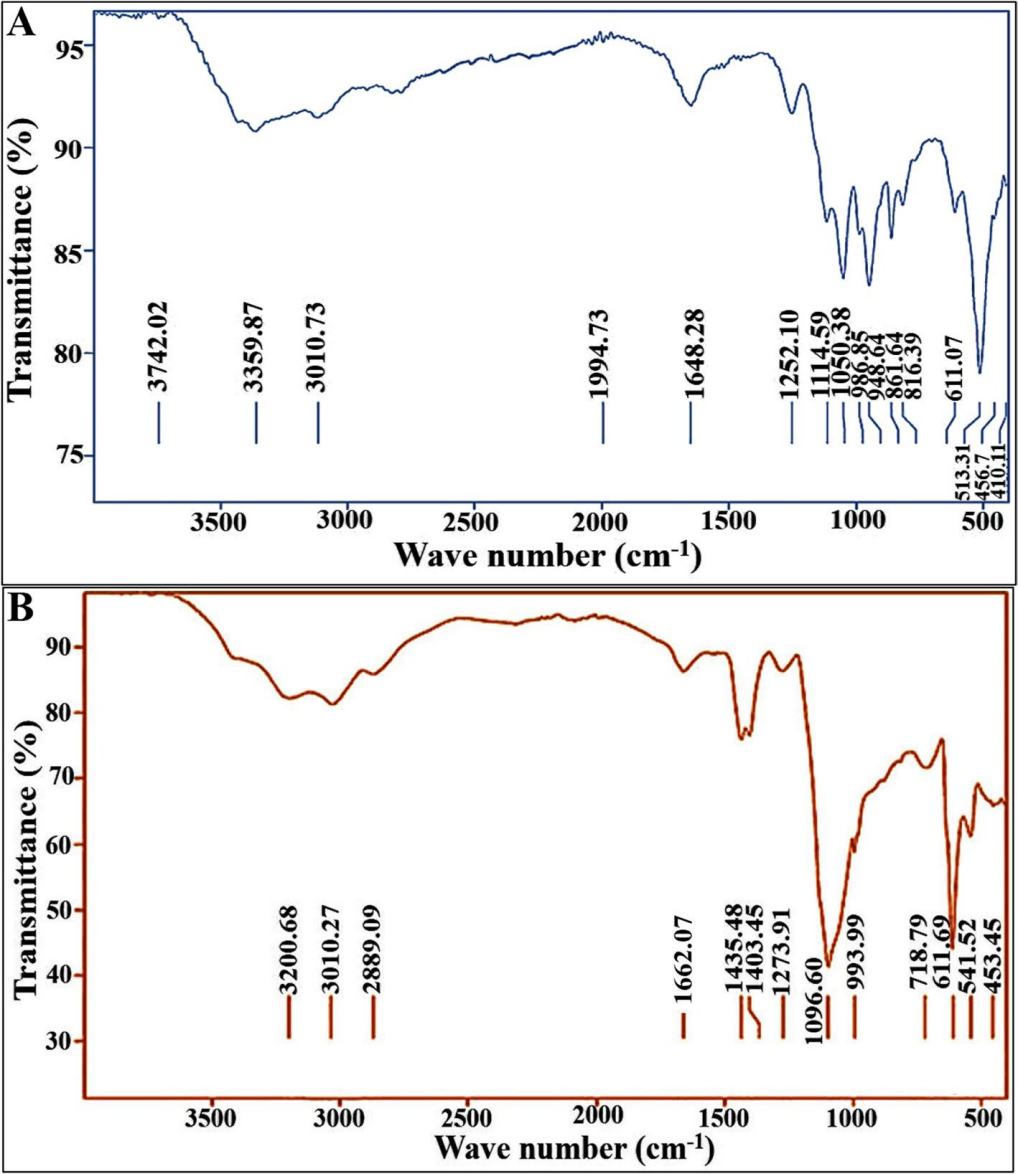
FTIR spectrum of oil samples from **(A) ***Alternaria* sp. **(B) ***Drechslera* sp

The acquisition of infrared spectra of lipids has been attained senior concern thank to providing opulent information on their chemical components, besides it is a fast and economical technique [33]. Fig. 2 demonstrates the FTIR spectrum of both examined SCOs. Generally, the peaks at wavenumbers of 3742 and 3359 cm^−1^ of A-OS and 3200 cm^−1^ of D-OS indicated the existence of hydrate hydroxyl group (-OH). Similarly, as signposted by Nandiyanto et al. [34], a broad absorption band in the range of 3650 and 3250 cm^−1^ represented the sign of hydrogen bond. Besides, the band at 3010 cm^−1^ in both SCOs samples pointed out to the C = CH- vibration originated from unsaturated fatty acids; interestingly, such band could be used for examining the degree of unsaturation in oils as referred by Shapaval et al. [35]. In the same sense, Nandiyanto et al. [34] stated that the bands above 3000 cm^−1^ are representative of unsaturated compounds. Accordingly, this could reflect the superior unsaturation of fatty acids in *Alternaria* sp. than that observed in *Drechslera* sp., as more peaks were detected in the area above 3000 cm^−1^, which harmonized with results of GC-MS that confirmed the higher unsaturation degree in A-OS comparing to D-OS.

Notably, the spectral bands at 2889 cm^−1^ detected in D-OS characterizes the C-H stretching vibrations of lipids [36]. Meanwhile, Nandiyanto et al. [34] mentioned that the double bond groups such as carbonyl (C = C) were present in the region of 1500–2000 cm^−1^. So, remarkably, both fungal patterns confirmed the presence of carbonyl (C = C) peaks as detected at 1994 and 1648 cm^−1^ of A-OS and 1662 cm^−1^ of D-OS. While the presence of spectral band at that regions reflected the presence of crystallizable FAs [33]. However, the signature of CH_2_ asymmetric bending and CH_2_ vibration could be detected by the presence of peaks at wave numbers of 1403 and 1435 cm^−1^ [33, 37]. Whereas, typical bands at 1252, 1114, 1050, 986 and 948 cm^−1^ of A-OS-FTIR pattern and also 1273, 1096 and 993 cm^−1^ of D-OS FTIR pattern referred to C-O stretching of phospholipids [38–40]. Regarding to the spectral bands at 718 and 611 cm^−1^ of D-OS and A-OS profiles, respectively, could be attribute to alkyne C-H bend and CH_2_ rocking vibration, respectively [34, 41]. Likewise, Forfang et al. [42] elucidated that biological material (e.g., carbohydrates, proteins and lipids) exhibits CH stretching vibrations due to the presence of -CH_3_ and -CH_2_. Arguably, based on the above mentioned characterization techniques, the results of FTIR were deemed as strong evidence for the presence of functional groups that were related to intracellular lipids, which were detected and quantified by GC-MS analysis; emphasizing hereby the obvious discrepancies between SCOs of *Alternaria* sp. and *Drechslera* sp. in the content and structures.

### Antibiofilm activity of SCOs

The presence of biofilm represents a serious threat to human health and surrounding ecosystem in various sectors. The biofilms injure medical equipment such as catheters, contact lenses, prosthetic devices, heart pacemakers, endoscopes, colonoscopes, dental plaques and dental irrigation units. Let alone their capability to invade human tissues causing severe infections [43]. However, the biofilms also adhere to food manufacturing equipment, air-conditioning units, petroleum pipelines and cooling towers, which symbolizes as evidences on industrial risk of biofilm. In the same sense, biofilms fixed themselves on external surfaces of marine vessels, water pipes, stones in a stream and sewage treatment plants, facilitating the accumulation of organic and inorganic materials with other organism such as algae, plants and protozoa in a phenomenon called biofouling. The problem of biofouling lies behind deteriorating the aqueous flow system with its fauna; causing the prevalence of microbial contamination and accelerated corrosion.

Against this backdrop, various mechanical removal approaches and chemical biocides were utilized to eliminate biofilms and prevent their hazard [44]. Nonetheless, the extensive use of antimicrobial agents generates multidrug resistance (MDR) phenomenon that led to ecological balance disruption and epidemic diseases. Therefore, modern insights are directed toward employing natural bioproducts as ecofriendly, biocompatible, safe and economic agents in defeating water/foodborne pathogens, which harmonized with aims of recent international events like COP28. Hence, the current investigation is undertaken to determine the in vitro antibiofilm activity of SCOs extracted from *Alternaria* sp. and *Drechslera* sp. against some MDR microbes-forming biofilm. This target was implemented through detecting the effect of SCOs on biofilm formation, viability, biochemical composition and hydrophobicity.

**Fig. 3:**
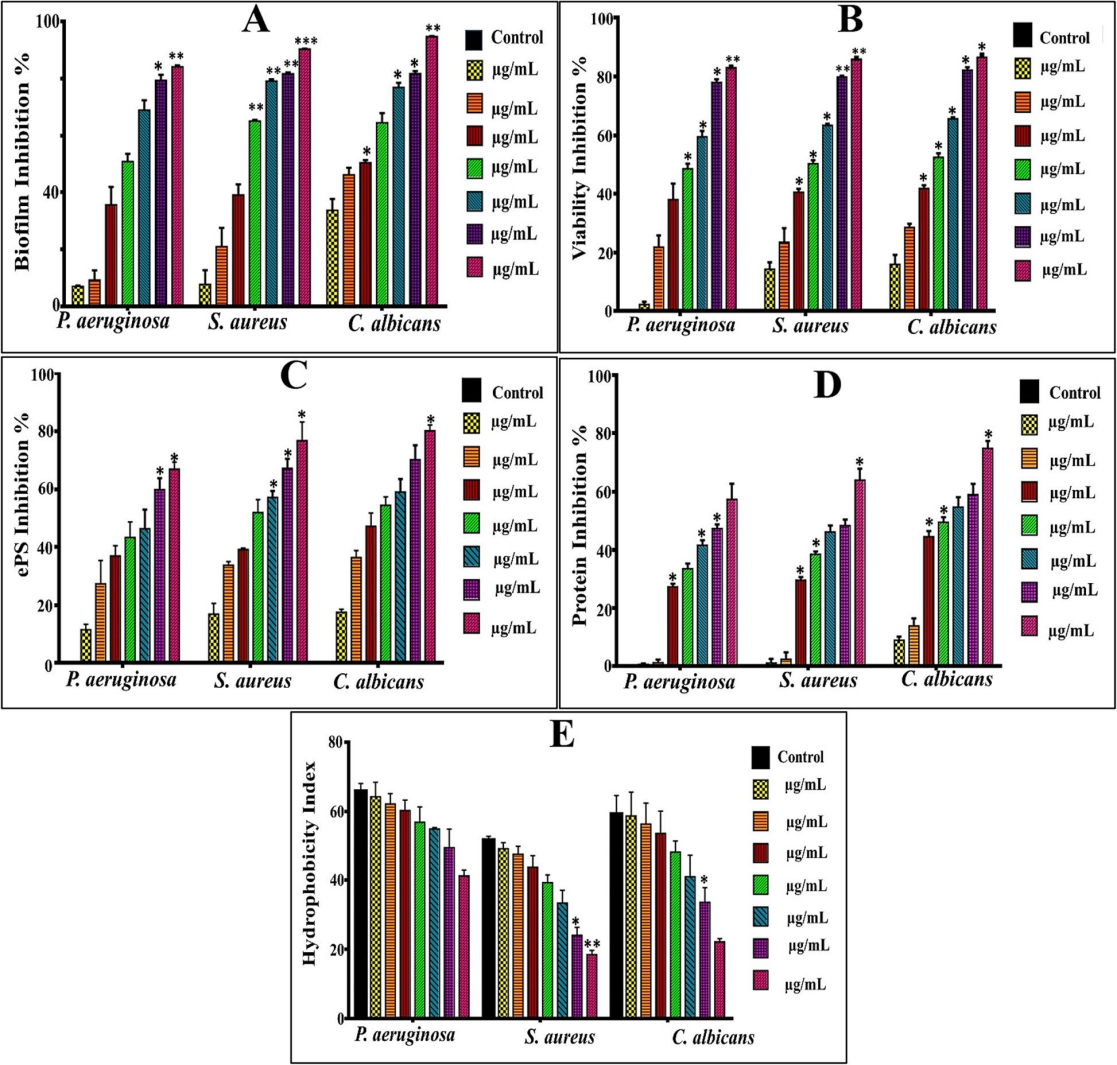
The Impact of A-OS on biofilm development by *P. aeruginosa, S. aureus *and* C. albicans*. **A**-Biofilm biomass suppression, **B**-Metabolic performance, **C**- EPS inhibition, **D**-Protein inhibition and **E**-Hydrophobicity inhibition. All values were expressed as mean ± SEM. Treatments at various doses were comparing to untreated control with significance at *P ≤ 0.05

**Fig. 4:**
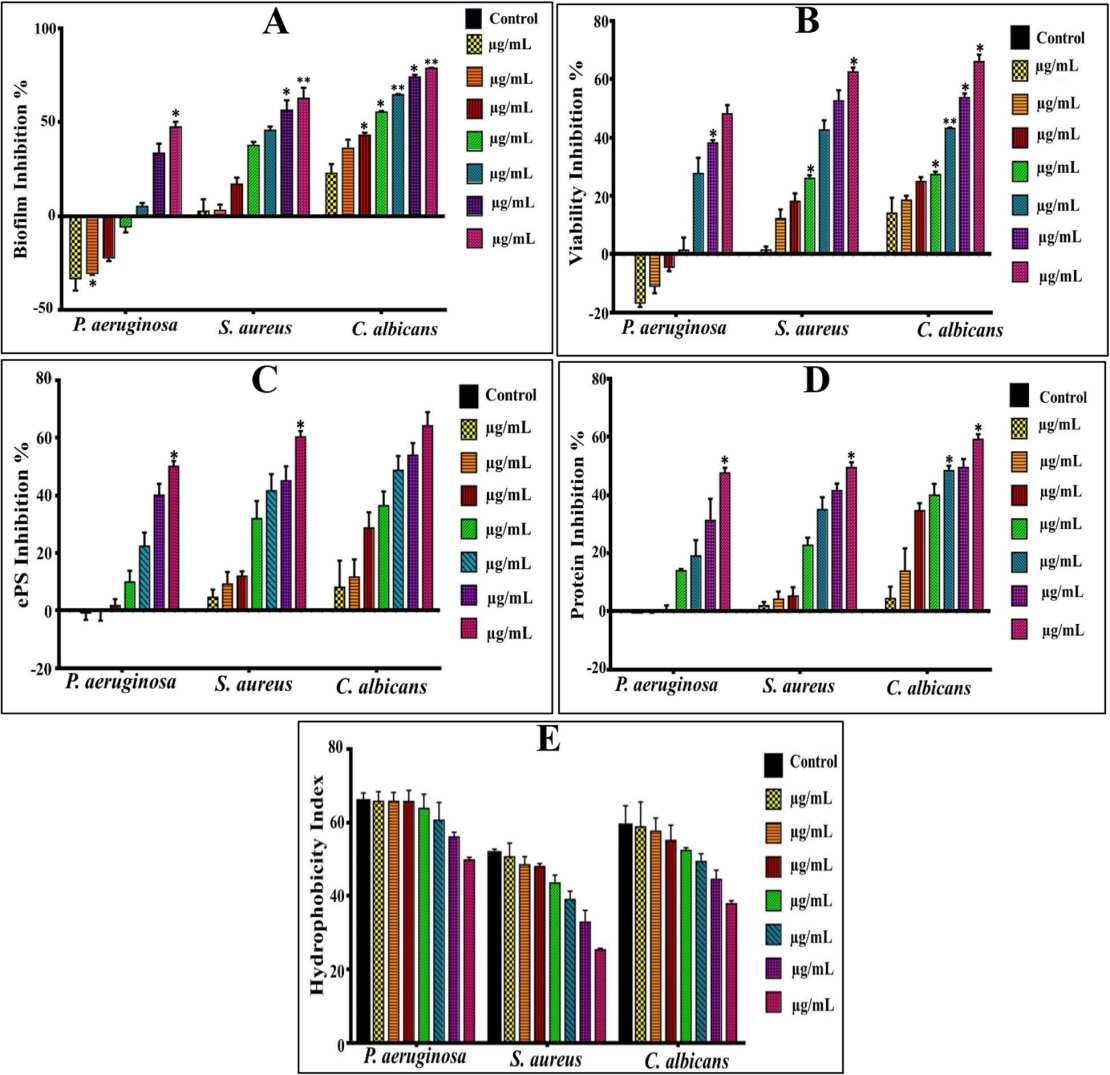
The Impact of D-OS on biofilm development by *P. aeruginosa, S. aureus *and *C. albicans. ***A**-Biofilm biomass suppression, **B**-Metabolic performance, **C**- EPS inhibition, **D**-Protein inhibition and **E**-Hydrophobicity inhibition. All values were expressed as mean ± SEM. Treatments at various doses were comparing to untreated control with significance at *P ≤ 0.05

In fact, *S. aureus*,* P. aeruginosa* and *C. albicans* were opted due to their ubiquitous occurrence and concomitance with nosocomial/community-acquired infections. Besides, they exhibit the capability to colonize vast array of surfaces either abiotic or cellular interfaces, which lead to significant environmental and health threats. Therefore, crystal violet assay (CV) was employed to detect the antibiofilm influence of different doses of SCOs (1–100 µg/mL), which deemed as reliable and facile assay in staining the biofilm biomass entirely [45, 46]. As observed in Figs. (3 and 4), the inhibitory patterns of both SCOs samples displayed significant differences (*P* ≤ 0.05) in the biofilm development after treatment, as unveiled by ANOVA. Besides, the inhibitory power of both samples showed notoriously variation against examined pathogens. Namely, SCOs of *Alternaria* sp. (A-OS) suppressed the growth of *P. aeruginosa* biofilm at concentrations ranged from 1 µg /mL to 10 µg/mL by 7.01 ± 0.3% to 50.85 ± 2.68%, respectively; while SCOs of *Drechslera* sp. (D-OS) enhanced the growth of *P. aeruginosa* biofilm by 33.41 ± 6.24% and 5.83 ± 2.83% at exact concentrations, respectively. Remarkably, the antibiofilm potency increased with elevation of applied doses of both examined oil samples. Wherein, the biofilm of *P. aeruginosa* was inhibited significantly (*P* ≤ 0.05) at 100 µg/mL of A-OS and D-OS by 84.10 ± 0.445 and 47.41 ± 2.83%, correspondingly. On the other hand, about 90.37 ± 0.065% and 62.63 ± 5.82% inhibition was observed for *S. aureus* biofilm at 100 µg/mL of both SCOs in the same order. Furthermore, a pronounced and significant (*P* ≤ 0.05) fungicidal potency was noticed in blocking the biofilm formation of *C. albicans* that reached to 94.96 ± 0.21% and 78.67 ± 0.23% upon treating with A-OS and D-OS (100 µg/mL), respectively. Generally, as inferred from these results, there is an inter-species variation phenomenon in a dose dependent performance exerted by the examined SCOs samples. In agreement with our results, Murugan et al. [47], found a variation in the biofilm growth of *Proteus* sp., *E. coli*,* Bacillus* sp. and *S. aureus*; assigning that to the differences in the physiological behavior of different microbial species. Seemingly, the cell wall architecture, microbial physiology with varied metabolic performance and uptake/regulation systems are considered being the fundamental parameters in managing the tolerance and susceptibility profiles among inter and intra-species of the microbes in their response to any antagonistic agent [48].

#### Effect of SCOs samples on biofilm metabolic activity

Actually, CV firmly stains the entire biofilm biomass, which includes polysaccharides in the mucilaginous mat conjugated with other biomolecules that are disseminated in an even manner on the live as well as dead cells surface. Subsequently, the overall metabolic performance of adhered microbial cells, which were treated with different concentrations of oil samples relative to untreated, was assessed calorimetrically using MTT assay. It is worth noting that tetrazolium salts (such as MTT (3-[4, 5- dimethylthiazol-2-yl]-2, 5-diphenyltetrazolium bromide), XTT (2,3-bis(2-methoxy-4-nitro-5-sulfo-phenyl)- 2 H-tetrazolium-5-carboxanilide) and TCC (2,3,5- triphenyl tetrazolium chloride) are frequently utilized in biological assays to investigate the viability of living cells. That occurs through enzymatic reduction of tetrazolium salt by the cellular NADH of metabolically active cells, which leads to the formation of colored formazan. Hence, different tetrazolium-based dyes were employed in various studies to determine biofilm viability in accompanying with other means like CV approach and microscale analysis [45, 46, 49].

Interestingly, both types of oil samples impacted on the viability of cells in the biofilm structure adversely, progressively and significantly (*P* ≤ 0.05) with increasing the doses of applied SCOs. Figs. (3 and 4) indicate that A-OS frustrated the propagation of active cell in *P. aeruginosa* biofilm matrix in all applied concentrations in the range of 2.35 ± 0.885% − 83.08 ± 0.235%. Conversely, D-OS flourished the growth of *P. aeruginosa* biofilm at low concentrations (1 µg/mL − 5 µg/mL) by the range of 16.72 ± 1.36% − 4.43 ± 1.36%; whereas, the viability of live cells curtailed to 48.12 ± 3.0% upon increasing the concentration to 100 µg/mL. Regarding to the biofilm of *S. aureus*, both oil samples inhibited the growth of active cells in linear concentration-dependent behavior; reaching to the maximum suppression by 85.9 ± 0.375 and 62.48 ± 1.52% for A-OS and D-OS, respectively at 100 µg/mL. In the same sense, the survival of *C. albicans* cells in biofilm network arrested by 86.54 ± 0.86% and 64.94 ± 1.48% under the treatment of A-OS and D-OS (100 µg/mL), respectively. As noticed, the results of biofilm inhibition were harmonized with that of metabolic activity. It is plausible to mention that viability and metabolic activity of all tested biofilm-forming pathogens correlated significantly with biofilm biomass (*r* ≥ 0.9, *P* = 0.00); reflecting hindrance impact of oil samples on active cells that are distributed within multilayer architecture of biofilm.

#### The effect of oil samples on biofilm’s carbohydrate and protein content

Carbohydrates or exopolysaccharides (ePs) and proteins represent the intrinsic constituents of EPS scaffold of the biofilm from both structure and function. As denoted by Gunn et al. [50] and Mosharaf et al. [51], the secreted proteins, adhesion proteins (e.g., lectins, Baplike proteins) and motility organelles configure the biofilm matrix proteins. However, galactose, mannose, glucose, arabinose, xylose, rhamnose, fucose, cellulose nanofibers, galacturonic acid and N-acetyl-glucosamine are the most abundant carbohydrates detected in slimy matrix of *S. aureus*, *Enterococcus faecalis*,* Klebsiella pneumoniae*, and *P. aeruginosa* [52]. As declared by Gunn et al. [50] the chemical constituents of the biofilm differ rely on the organism and was influenced by environmental parameters. Intriguingly, such specific components control biofilm integrity, maintain biofilm stability, configure its morphology, mediate cell-cell signaling, contribute in cell colonization/adherence and preserve its cells from adverse external stressors [23, 51].

Figure (3) depicts the effect of A-OS on biofilm content of carbohydrates or ePS, which diminished from 7.515 ± 0.13, 5.541 ± 0.147 and 6.59 ± 0.33 mg/mL in control untreated samples of *S. aureus*,* P. aeruginosa*, and *C. albicans* biofilms to 3.61 ± 0.34, 3.14 ± 0.335 and 2.99 ± 0.19 mg/mL at 10 ug/mL of A-OS, respectively; representing by such way inhibition percentages of 51.87 ± 4.55, 43.26 ± 5.9, and 54.5 ± 2.84%, respectively. However, the same concentration of D-OS reduced carbohydrate content of *S. aureus*,* P. aeruginosa*, and *C. albicans* biofilms to 5.100 ± 0.47, 4.98 ± 0.22, and 4.20 ± 0.335 mg/mL by 32.09 ± 0.536, 9.94 ± 4.04, and 36.23 ± 5.04% inhibition percentages, respectively (Fig. 4). In the same context, 10 ug/mL of A-OS reduced protein content of *S. aureus*,* P. aeruginosa* and *C. albicans* biofilms from 9.64 ± 0.095, 9.75 ± 0.065 and 9.34 ± 0.1 mg/mL in the control untreated samples to 5.92 ± 0.11, 6.94 ± 0.17 and 4.72 ± 0.16 mg/mL, which symbolize 38.54 ± 0.94, 33.47 ± 1.74 and 49.43 ± 1.69% inhibition percentages, respectively. Whereas, D-OS (10 ug/mL) diminished protein content to 7.44 ± 0.255, 8.40 ± 0.065 and 5.61 ± 0.37 mg/mL causing 22.81 ± 2.63, 13.84 ± 0.59 and 39.92 ± 3.89% inhibition.

Notably, significant and dramatic changes were observed in both contents upon elevating the concentrations till reached to the highest values at 100 ug/mL. Wherein, such concentration of A-OS caused in lowering the carbohydrate/ePS content of *S. aureus*,* P. aeruginosa* and *C. albicans* biofilms by 76.54 ± 7.04, 66.81 ± 2.51 and 80.54 ± 1.77%, respectively. While the carbohydrate/ePS reduction percentage reached 60.22 ± 2.02, 50.03 ± 1.95 and 64.26 ± 4.7% by D-OS for *S. aureus*,* P. aeruginosa* and *C. albicans* biofilms, respectively. Regarding the protein content, it lessened significantly to 63.94 ± 3.89, 57.27 ± 5.43 and 74.87 ± 2.49% upon applying 100 ug/mL of A-OS; however, D-OS (100 ug/mL) reduced it to 49.39 ± 1.96, 47.61 ± 2.03 and 59.07 ± 1.78% for *S. aureus*,* P. aeruginosa* and *C. albicans* biofilms, respectively.

#### Effect of oil samples on biofilm hydrophobicity

The adhesion capability of microbial cell deemed as intrinsic property to colonize any substrate and boost biofilm lifestyle easily. Interestingly, cell surface traits in the formed of ePS and hydrophobicity are decisive parameters that manage the entire adhesion process. In addition, surface characteristics and ambient environmental conditions rule the adhesion of the cells with the surface through number of interactions like hydrophobic, van der Waals and electrostatic. Remarkably, the cells with higher biofilm forming capacity possess higher hydrophobic nature that leads to potent adhesion and vice versa [53]. Given that the biofilm-forming cells with hydrophobic characteristics exhibit affinity to hydrocarbons (e.g., hexadecane, octene, xylene, etc.), the cells retained in the organic phase; generating low turbidity of aqueous phase and by such method (i.e., MATH), the hydrophobicity nature of the biofilm is detected [54].

In the current study, the hydrophobicity index (HI) recorded 65.72 ± 2.3, 51.61 ± 1.12 and 59.12 ± 5.43% for *P. aeruginosa*,* S. aureus* and *C. albicans* respectively; denoting a higher hydrophobicity property of *P. aeruginosa* than that exhibited by *S. aureus* and *C. albicans*. Upon applying different concentrations (10–100 µg/mL) of both oil, a noticeable reduction in HI was shown (Figs. 3 and 4); implying progressively transition to hydrophilicity state, which reached to the maximum at the highest applied doses. Wherein, A-OS (10 µg/mL) lessened hydrophobicity to 64.18 ± 4.23, 49.18 ± 1.73 and 58.62 ± 6.94% for *P. aeruginosa*,* S. aureus* and *C. albicans*, by 1.54 ± 4.23, 2.43 ± 1.73 and 0.498 ± 6.91% inhibition percentage in the respective order. However, A-OS (100 µg/mL) switched *S. aureus* and *C. albicans* biofilms to weak hydrophobicity state (i.e., became hydrophilic) by recording 18.5 ± 1.22 and 22.18 ± 0.94% HI, respectively; implementing 33.1 ± 1.22 and 36.94 ± 0.94% inhibition. While, the HI of *P. aeruginosa* biofilm altered to be moderate by recording 41.32 ± 1.65% and inhibition percentage recorded 24.04 ± 1.65%. On the other hand, D-OS (10 and 20 µg/mL) insignificantly enhanced the hydrophobicity of *P. aeruginosa* biofilm by 0.046 ± 2.55 and 0.032 ± 2.364%, respectively. Whereas, at 100 µg/mL inhibited its hydrophobicity by 15.96 ± 0.74%; maintaining HI in the potent region by recording 49.78 ± 0.74%. Regarding *S. aureus* and *C. albicans* biofilms, D-OS promoted their hydrophilic affinity in a dose-dependent behavior, reaching to the maximum at 100 µg/mL by recording 25.34 ± 0.422 and 37.89 ± 0.826%, correspondingly, which all remained in the moderate phase of hydrophobicity. Generally, albeit distinct structural variation in cells surface among the examined strains in our study, both oil samples substantiated their efficacy in influencing on hydrophobicity adversely. It is important to highlight that hydrophobicity reflects the microbial attachment or adhesiveness ability, which varies even from strain to strain and influenced by microbial age, microbial surface charge and growth medium [55]. In study performed by Kim et al. [56], 10 µg/ml of antibiofilm FAs (e.g., tricosanoic acids, palmitoleic, myristoleic acid, lauric acid, stearic, heptadecanoic and α-linolenic) reversed the biofilm of *Cutibacterium acnes* from hydrophobic region to hydrophilic region (hydrophobic index < 20%) simultaneously with biofilm inhibition, which agreed with our results.

**Fig. 5:**
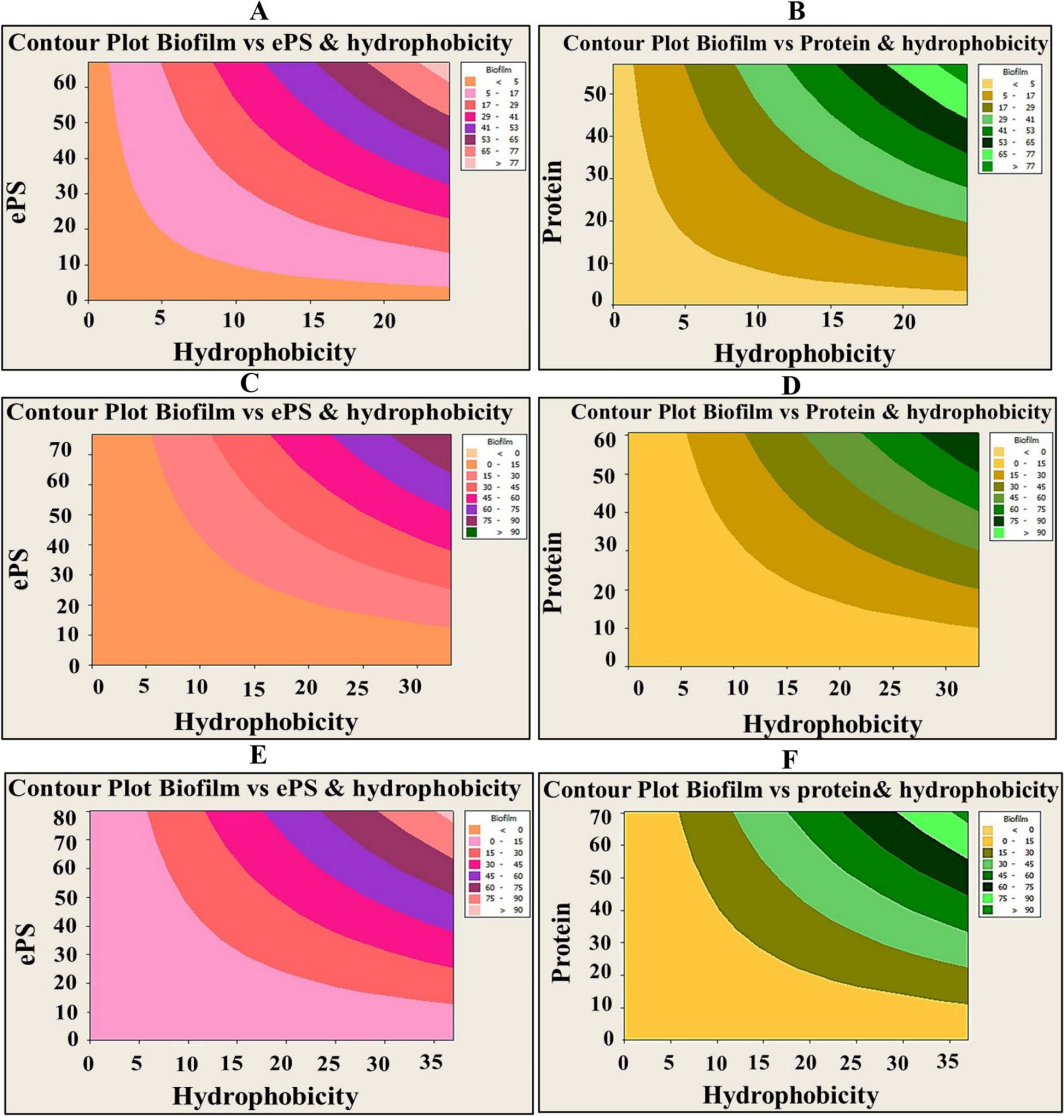
Contour plot showing the correlation of biofilm suppression by A-OS versus EPS inhibition (left panel) and protein inhibition (right panel) with cell surface hydrophobicity. The diagram was plotted by Minitab 14 software. Different colors elucidate different levels of biofilm suppression. **A** & **B** - P. aeruginosa, **C** & **D**- S. aureus and **E** & **F**- C. albicans

**Fig. 6:**
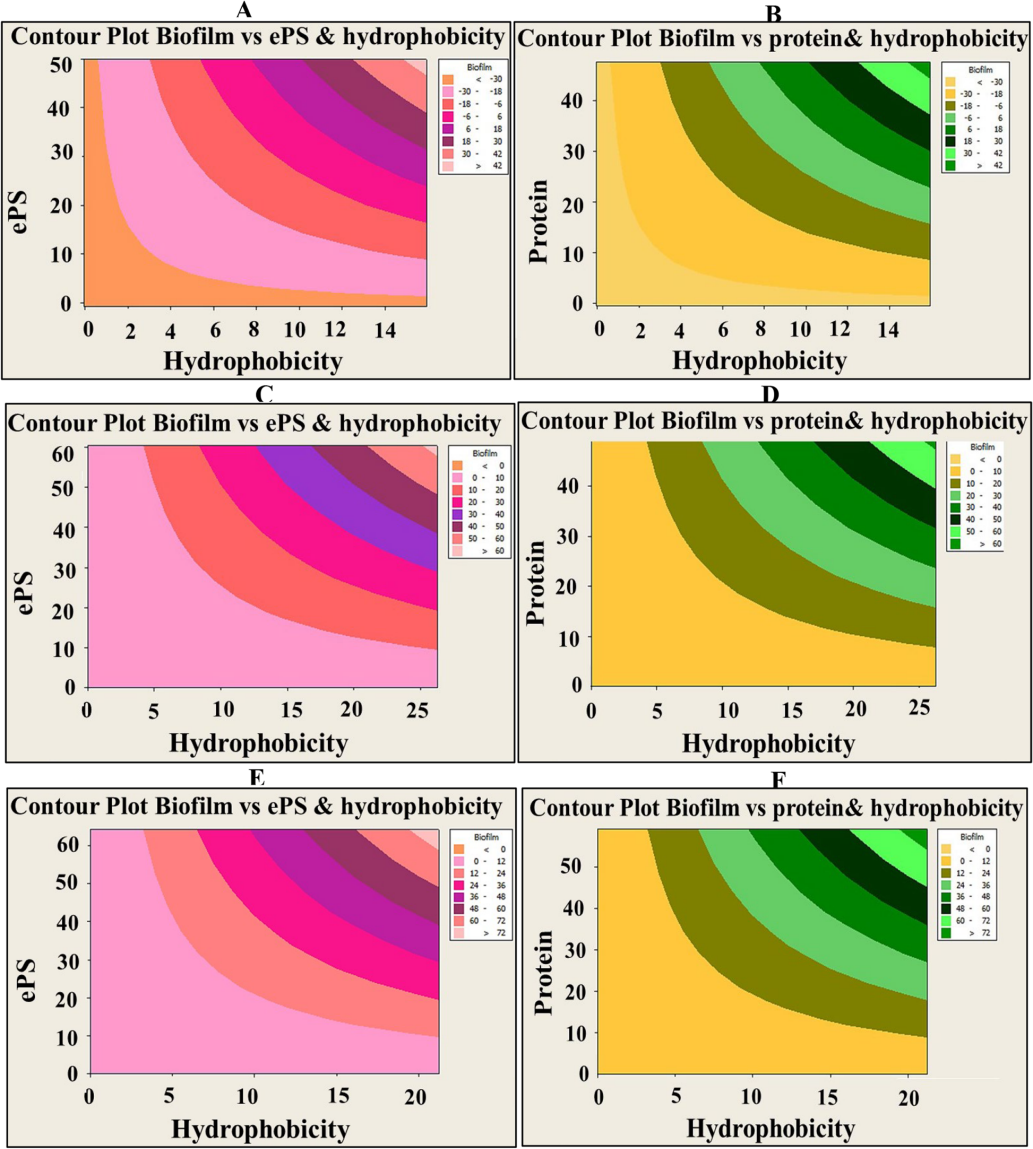
Contour plot showing the correlation of biofilm suppression by D-OS versus EPS inhibition (left panel) and protein inhibition (right panel) with cell surface hydrophobicity. The diagram was plotted by Minitab 14 software. Different colors elucidate different levels of biofilm suppression. **A** & **D** -* P. aeruginosa, ***B** & **E**-* S. aureus *and **C** & **F**- *C. albicans*

Intriguingly, the results of the current investigation declared the existence of significant positive correlation between all examined variables (i.e., inhibition of biofilm, protein, ePS, viability and hydrophobicity) with SCOs concentrations, as signified by Pearson’s correlation coefficients (Table 2) (Figs. 5 and 6). Wherein, oil samples influenced negatively on the biofilm development through modulating microbial-surface interactions, in particular hydrophobic interactions through impacting on surface-associated exopolysaccharides and proteins. In consistent with our results, Pompilio et al. [57] attributed the higher hydrophobicity of *Stenotrophomonas maltophilia* biofilm to its higher exopolysaccharides content, which was positively correlated with biofilm development. Also, Mu et al. [58] manifested and explained the same finding in *S. epidermidis* biofilm. Otherwise, several reports documented the independence of biofilm formation on hydrophobicity [53, 59]. Nonetheless, there is a consensus among all studies regarding that the cell surface properties and overall physiological properties of microbes govern the process of biofilm development and maturation. Noteworthy mention that the hydrophobicity is fostered by the action of microbial appendages (e.g., pilli, fimbriae, fibrils, etc.) that scattered on the cell surface. Such organelles contain hydrophobic amino acid residues that expedite noncovalent attachment of the cells on any substratum [54, 55]. However, exopolysaccharides facilitate irreversible adhesion and sheltering the developed cells within the backbone of biofilm [23, 52].

**Table 2:** Representing the correlation between biofilm inhibition with other studied factors (i.e., viability, ePS, protein and hydrophobicity) by the action of SCOs

Biofilm type	Examined parameter	Pearson’s correlation coefficients (R^2^) (*P*-value)							
		SCOs-1				SCOs-2			
		Biofilm	Viability	ePS	Protein	Biofilm	Viability	ePS	Protein
***P. aeruginosa***	**Viability**	0.983 (0.00)				0.961 (0.00)			
	**ePS**	0.954 (0.00)	0.985 (0.00)			0.941 (0.00)	0.971 (0.00)		
	**Protein**	0.983 (0.00)	0.968 (0.00)	0.943 (0.00)		0.934 (0.001)	0.95 (0.00)	0.987 (0.00)	
	**hydrophobicity**	0.926 (0.001)	0.942 (0.00)	0.928 (0.001)	0.911 (0.002)	0.928 (0.001)	0.942 (0.00)	0.984 (0.00)	0.986 (0.00)
***S. aureus***	**Viability**	0.981 (0.00)				0.976 (0.00)			
	**ePS**	0.97 (0.00)	0.984 (0.00)			0.986 (0.00)	0.982 (0.00)		
	**Protein**	0.979 (0.00)	0.975 (0.00)	0.941 (0.00)		0.984 (0.00)	0.982 (0.00)	0.992 (0.00)	
	**hydrophobicity**	0.932 (0.001)	0.968 (0.00)	0.931 (0.001)	0.951 (0.00)	0.947 (0.00)	0.977 (0.00)	0.97 (0.00)	0.973 (0.00)
***C. albicans***	**Viability**	0.975 (0.00)				0.958 (0.00)			
	**ePS**	0.984 (0.00)	0.984 (0.00)			0.969 (0.00)	0.973 (0.00)		
	**Protein**	0.937 (0.001)	0.965 (0.00)	0.965 (0.00)		0.964 (0.00)	0.933 (0.001)	0.986 (0.00)	
	**hydrophobicity**	0.879 (0.004)	0.932 (0.001)	0.886 (0.03)	0.88 (0.004)	0.891 (0.003)	0.971 (0.00)	0.95 (0.00)	0.9 (0.002)

Based on the previous results, it is conspicuous that the antibiofilm potency of SCOs samples appeared more evident against *S. aureus*. That could be attributed to its physiological and metabolic sensitivity, besides the nature and architecture of its cell wall, which seemed to contribute intrinsically in its susceptibility. Namely, the hydrophilic nature of gram-positive bacteria’s cell wall with their low content of lipids (1–4%) trigger the adsorption and penetration processes of exogenous materials interiorly easier. In contrast, the more complex structure of gram-negative bacterial cell wall with its abundant hydrophobic moieties (11–22% lipid content) serves as potent entry barrier toward detergents and hydrophobic molecules, hindering by such way the internal transportation of SCOs [60]. Strikingly, the superior biocidal potency of fatty acids, especially those of longer chain FAs (i.e., ≥ C12) against gram-positive bacteria such as *B. subtilis*,* Micrococcus luteus*,* Propionibacterium acnes*, *Listeria monocytogenes* and *Clostridium difficile* was reported tremendously by several research groups [56, 61–65], which coincident with our results. Additionally, Shukla et al. [62] reported that gram-negative bacteria displayed more resistance to medium- and long-chain FAs than gram-positive bacteria, which also agreed with our results; however, gram-negative bacteria were more susceptible to FAs with C6 or less in their chain. In the same context, [60] opined that yeast cells are more susceptible to medium chain-FA (i.e., C8-C12). Otherwise, [63, 66–68] stated the mycocidal potency of long chain-FA. Herein, both SCOs samples of the present study exhibited promising fungicidal performance versus *C. albicans*, which agreed that found by previously mentioned studies. Hereby, both oil samples could be harnessed in thwarting candidiasis in immunocompromised patients following secondary pneumonia infections conjugated with COVID-19.

Arguably, the biofilm development by *P. aeruginosa*,* S. aureus* and *C. albicans* was dose-dependently impeded by both oil samples, which could be ascribed to the intensification of detergent traits of FAs on account of their amphipathic nature, which could be bacteriostatic at lower concentrations and bactericidal at higher concentrations [56, 69, 70]. Subsequently, it is imperative to shed the light about the antagonistic strategy followed by oil against examined pathogens. However, as referred by Cepas et al. [66] and Kumar et al. [71] the precise biocidal strategy followed by FAs is obscure and undefined. Wherein, FAs frustrate microbial growth and biofilm development through violating multiple cellular targets nonspecifically, which seems being characteristic and should be invested in enfeebling drug resistance phenomena (MDR) [72].

The detrimental effects of FAs against treated biofilm commences with the cell membranes, which considered being the prime target in the multi-step process of biofilm formation. Wherein, FAs incorporated on the hydrophobic moieties of cell phospholipid layer causing membrane solubilization, integrity destabilization and apertures creation, either transient or permanent. That in turn elevated cell permeability level and cellular leakage. Besides, FAs subvert the nutrient uptake system, electron transport chain and oxidative phosphorylation which are the chief membrane-located processes. Additional presumptive scenario could be exerted by oil in the context of membrane damage is suppressing the functionality of membrane-associated proteins, which mediate fatty acid biosynthesis in the plasma membrane. Remarkably, Firoozabad et al. [73] demonstrated that the destructive effect of FAs on *S. aureus* biofilm was triggered through the inactivation of enzyme that is responsible for the fatty acid elongation step, which is enoyl-acyl carrier protein reductase (Fabl). On the other hand, Cepas and coauthors [66] assigned the inhibition of *P. aeruginosa* and *C. albicans* biofilms to the inhibition of Fabl by the action of FAs. Similarly, Lee et al. [67] attributed the anticandida potency of FAs to the hindrance of ergosterol production, which regulates fungal membrane synthesis and reinforces its structural. On the other hand, the treatment with FAs could enhance autolysis process by inducing autolytic enzyme in lieu of massive solubilization of the cell membrane by the surfactant-like action of FAs as suggested by Tsuchido et al. [74].

Interestingly, Bintari and Risandiansyah [75] scrutinized in silico the effect of FAs extracted from *Cladophora* sp. in dampening the activity of peptide deformylase in *P. aeruginosa*,* E. faecium*,* E. coli*,* and S. aureus*, which catalyzes protein maturation process. Meanwhile, FAs impair ePS production, fimbriae/pili formation and hydrophobicity, which thereafter frustrate motility and adhesion to substrates; ultimately block the microbial colonization and ruin irreversible aggregation and attachment. The finding by research groups of Kim et al. [56], Nicol et al. [76] and Kim et al. [77] supported these deleterious impacts. On genetic level, FAs drastically repressed DNA supercoiling/replication and down-regulated different genes such as hla, HWP1, CHT4, csgAB, fimH, flhD, motB, luxRS and NorA, which encode proteins responsible for alpha-hemolysin production, hyphal development, chitinase production, fimbriae synthesis, motility, quorum-sensing and efflux pump [63, 71, 77]. Additionally, anti-quorum sensing activity was detected for FAs in quenching the communication signaling and quorum sensing system among biofilm cells and with external environment [66, 76]. As referred by Nicol et al. [76], FAs inhibited the biofilm of *Acinetobacter baumannii* up to 38% through reducing quorum sensing regulator AbaR, which influenced adversely on communication signals among biofilm cells.

Taken together, the overall data revealed the superior antibiofilm potentiality of A-OS in defeating all examined types of biofilm-forming pathogens. That could be attributed to the profusion of USFAs (62.18%) comparing to D-OS, which contained 53.15% (Table 1-Fig. 1). Our finding is in line with the majority of the previously published investigations who declared the lower activity or bacteriostatic performance of SFAs [69, 78]. While USFAs with the same length carbon backbone exhibit influential potency in deteriorating biofilm development and obstructing the viability of its cells. Such potency of USFAs could be ascribed to their instability, propensity to oxidation and binding non-specifically to target sites like proteins [69, 78]. Otherwise, Khalilova et al. [79] demonstrated the remarkable antibacterial and antifungal properties of SFAs relative to USFAs, especially capric C10:0, lauric C12:0 and palmitic C16:0 acids.

Comparisons among studies are entangled and controversial due to the differences in the applied FAs, the treated microbe or even the altogether treatment process [69]. Notably, the nature of FAs (i.e., acid or its derivatives such as methyl, ester form, etc.), the applied dose and their solitary or combined case were deemed as the substantial features concerning FAs entity. However, the microbial factor includes microbial species types, isolation source, microbial cell load, microbial phase (i.e., planktonic or aggregated biomass), microbial metabolic activity and microbial physiology (i.e., sensitive or persister), which are undoubtedly differ among microbial genera till strain level. While the contact time, pH value and ionic strength of treatment milieu represent other influential treatment circumstances. All these reasons collectively could definitely influence on biocidal activity of applied FAs to different extends [60, 80]. Nevertheless, the molecular structure and shape of FAs remain the driving force that control the magnitude toxicity of FAs. Precisely, the carbon chain length, number of unsaturation and the geometric configuration around the double bond are the conclusive parameters that correlated proportionally to FAs antagonistic potentiality. As stated by McGaw et al. [60], Desbois and Smith [69] and Kim et al. [56], FAs that possess more carbon atoms (i.e. C16, C18, etc.) in their carbon backbone are more potent antimicrobial agents than those contained 10 or 12 carbons in their chain. Moreover, USFAs, properly PUFAs followed by MUFAs, manifested characteristic biocidal functionalities than SFAs [69]. In this regard, Feldlaufer et al. [78] found that palmitic acid (C16) didn’t show any inhibitory action against pathogens comparing to its unsaturated counterpart (i.e., palmitoleic acid (C16:1); confirming that FAs with 3, 4 or 6 double bonds display more antimicrobial activity. Strikingly, FAs stereochemistry also deemed as limiting factor, namely, the *cis-isomer* is highly active relative to *trans* configuration [60, 78]. That could be ascribed to the resemblance in the structure between SFAs and *trans* orientation USFAs [69]. In this sense, Feldlaufer et al. [78] documented that the introduction of double/multiple bonds, especially with FAs more than 14 carbon atoms in *cis-* orientation, plays imperative role in elevating antibiotic-like behavior. On the other hand, McGaw et al. [60] reported that SFAs with lower-chain length, MUFA and PUFAs with longer-chain structure exhibit considerable antimicrobial activity.

Remarkably, as indicated by the chromatographic profile (Fig. 1; Table 1), A-OS contained ample amount of omega (ω) fatty acids such as ω-5 (myristoleic acid, C14:1, 0.52%), ω-7 (palmitoleic acid, C16:1, 4.49%) and ω-9 (oleic acid C18:1, 24.78%). However, the predominance of ω-3 (cis-5,8,11,14,17-eicosapentaenoic, C20:5, 0.65%; Cis-4,7,10,13,16,19-docosahexaenoic, C22:6, 0.64% and Cis-11,14,17-Eicosatrienoic C20:3 0.60% ) and ω-6 (linolenic acid, C18:3, 22.67% and γ-linolenic acid, C18:3, 4.22%) contributed intensively in its robust effectiveness. It is worthy mention that ω-3 and ω-6 are frequently utilized as alternative food additives and therapeutic agents to maintain body homeostasis and enhancing the immune system, by the virtue of their functional values in lipid metabolism, antioxidant signaling pathway regulation and inflammatory processes modulation. Besides, their role in diabetes type two, ulcerative colitis, cardiovascular disease, Crohn’s disease, hypertension, alzheimer’s disease and cancer therapy couldn’t be neglected [62, 68, 73]. Meanwhile, their potency in defeating various microbial genera as antibacterial, antifungal or antibiofilm was also determined in various investigations [68, 79].

In the study conducted by Cepas et al. [66], the authors detected the inhibitory limit of linolenic acid by 1, 32 and 64 mg/mL against *C. albicans*,* S. aureus* and *P. aeruginosa*, respectively. Also, he and coauthors found that more than 250 mg/mL of stearic acid, γ-linolenic acid, arachidonic acid and palmitoleic acids were required to utterly block the growth of *E. coli*. While, 0.02 mg/mL of palmitoleic acids inhibited the biofilm of *A. baumannii* by 38% [76]. Comparing our results with previous studies and others, reflects the advantageous and promising properties of our SCOs that dampened biofilm activity, their cell viability, biochemical constituent and their hydrophobicity in sensible doses. That could be assigned to the synergistic effect mediated by the combination of SFAs, MUFAs and PUFAs collection. Strikingly, the naturally derived lipids from herbal and microbial sources share this common phenomenon. Namely, the presence of mixture of FAs that compete mutually on more than target site could implement their hostile functionality in prominent way. The results of the present research concurred with that reported by Dia and Jacoeb [81], Shukla et al. [62], Balkrishna et al. [72] and Panjaitan et al. [80]. In the context of synergistic activity, several reports accentuated the uplifting of antibiotic-like performance of FAs upon conjugating several FA together or supplementing with antibiotics, bacteriophages, organic compounds, enzymes and edible plant essential oils [63, 82–86].

### Safety and anticancer activities of oil samples

**Fig. 7:**
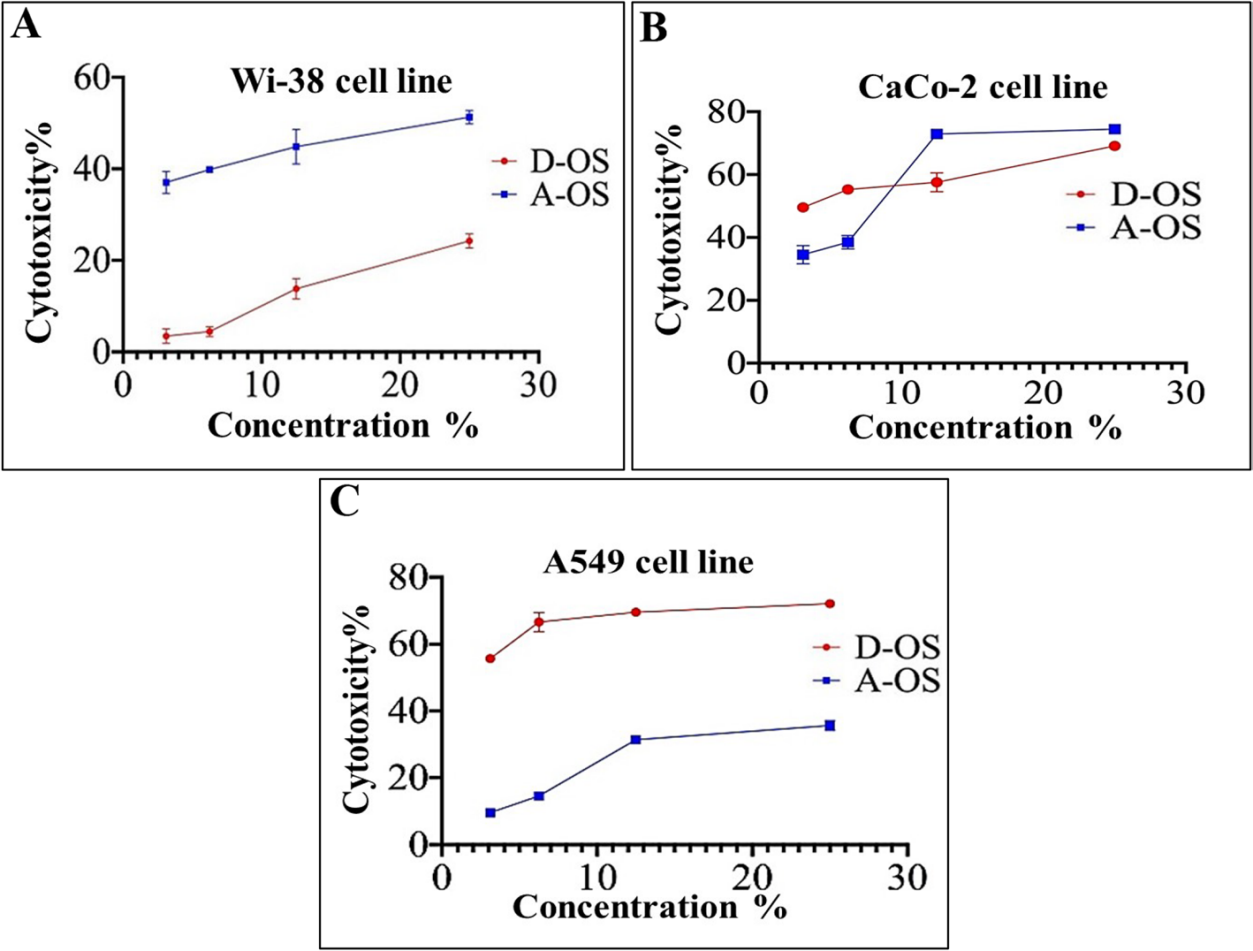
Cytotoxicity results of the oil samples on noncancerous (**A**) and cancerous cell lines (**B** and **C**)

**Fig. 8:**
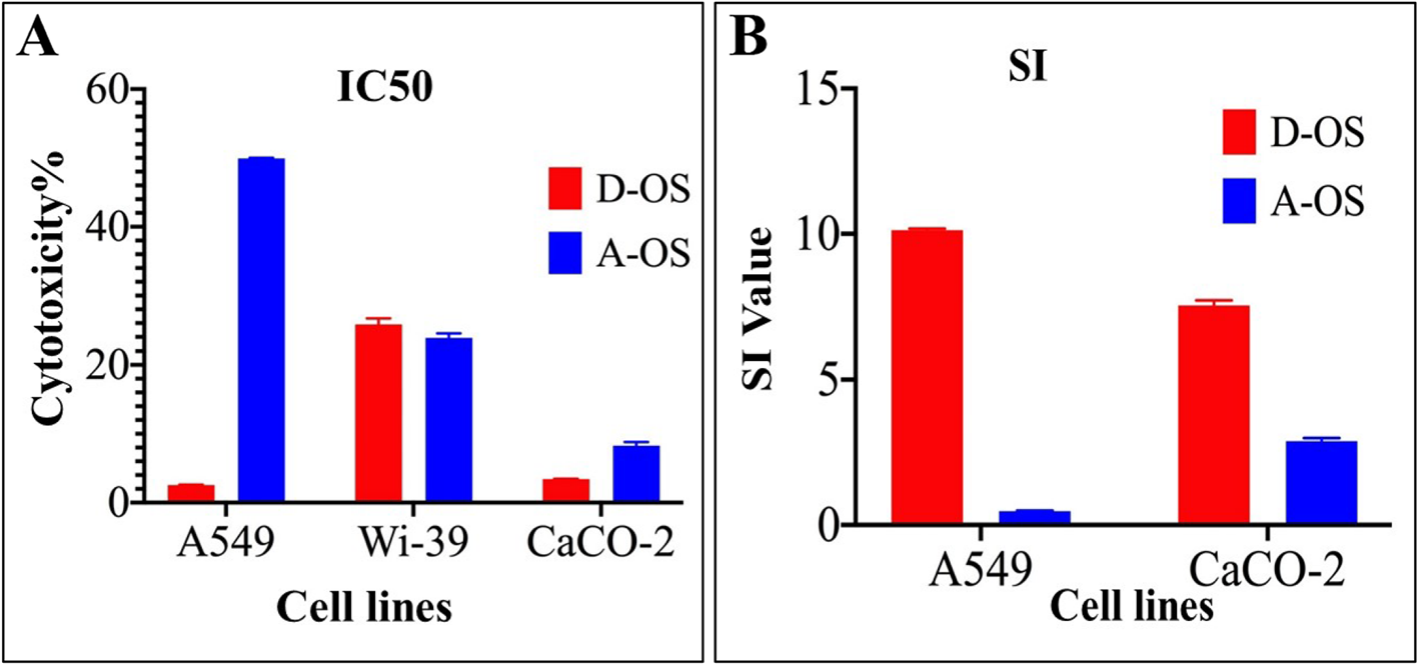
The IC50 values of the oil samples on the non-cancerous and cancerous cell lines (**A**), selectivity index (SI) against cancer cells (A549 and CaCo-2) (**B**), and morphological variations in the treated cancer cell lines relative to the untreated cells (**C**)

The cytotoxicity results of oil samples on the non-cancerous cell line (Wi-38) indicated that the D-OS was safe treatment on the viability of these non-cancerous cells with IC50 25.8% followed by A-OS with IC50 value of 23.86% (Figs. 7A and 8). As a second-leading reason of death, the continuous amending in cancer treatment modalities is intensively implementing. The most widely options of cancer treatment for many decades are surgery, chemotherapy and radiation therapy, in a single treatments or in combination (Debela et al., 2021). Actually, pain and fatigue are the mainly harmful side effects of conventional therapies modality, besides, dosage selection difficulty, rapid drug metabolism, cytotoxicity and lack of tumor-specific treatments [87, 88]. Henceforth, utilizing fungal bioactive compounds such as SCOs considers being promising, complementary and alternative therapy to reduce cancer complications and improve the therapeutic efficacy [89].

**Fig. 9:**
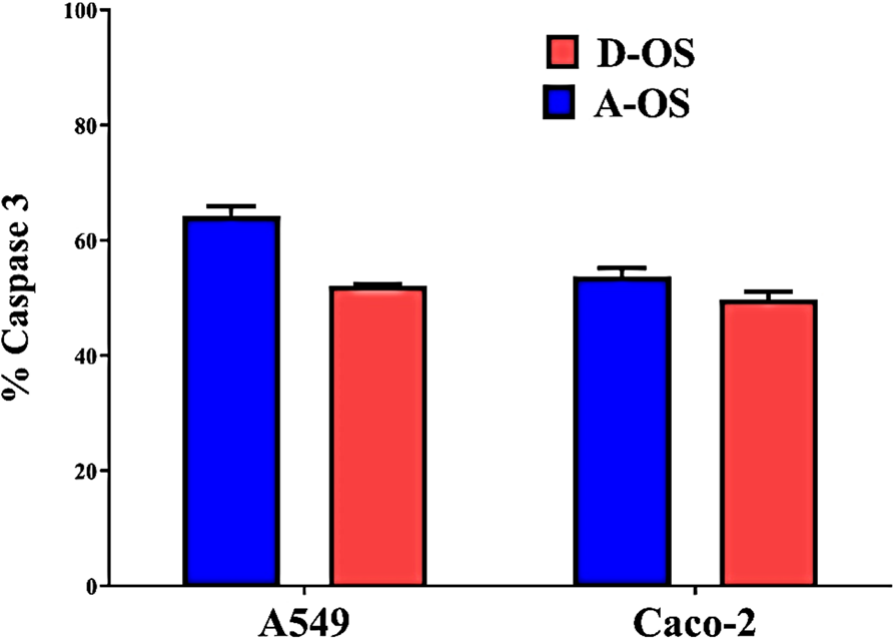
Caspase 3 activation percentages in D-OS and A-OS treated A549 and Caco-2 cells

**Fig. 10:**
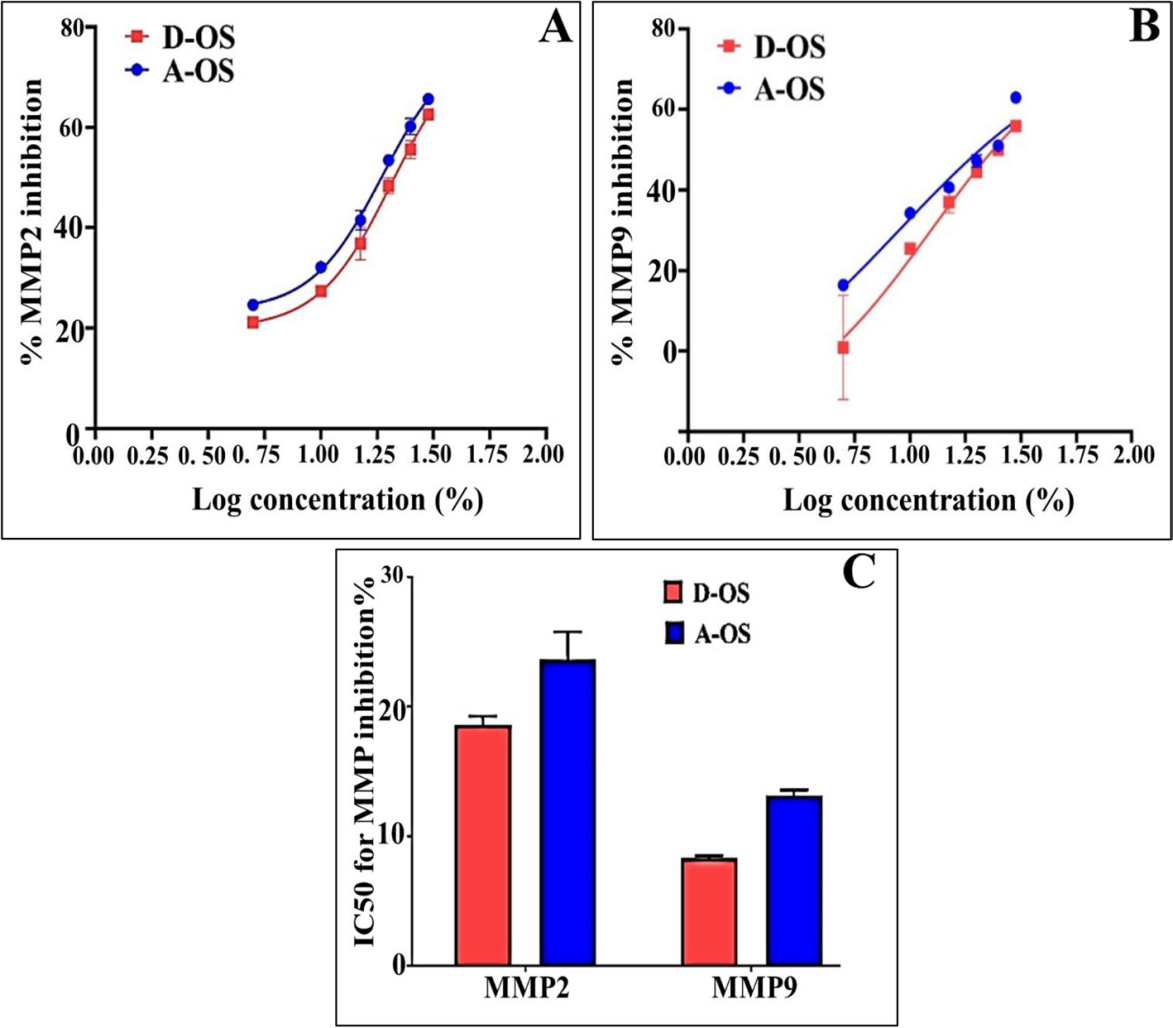
Dose response curves of A-OS-and D-OS for (**A**) matrix metalloproteinase MMP2 and (**B**) MMP9 inhibition as well as (**C**) their IC50 values

Concerning with the anticancer effects of the A-OS and D-OS, the MTS assay protocol showed that the oil sample D-OS is the most potent cytotoxic agent against both A549 and CaCo-2 cell lines with IC50 values of 2.55 and 3.425%, respectively (Figs. 7B and 8) and SI values of 10.1 and 7.5, respectively (Fig. 8). Furthermore, A-OS showed a potent effect against CaCo-2 cell line with IC50 8.275% (Fig. 8A) and SI value of 2.88 (Fig. 8B). D-OS was safer than A-OS on the viability of normal cells (Wi-38) with IC50 value that was 10-fold higher than its corresponding concentrations for inhibiting 50% growth of CaCo-2 and A549. Meanwhile, another oil sample (i.e., A-OS) had a higher IC50 for A549 growth inhibition than its cytotoxic dose on Wi-38. This declares that D-OS is more selective cytotoxic oil than A-OS against both studied cancer cell lines. Importantly, A-OS activated caspase 3 by 64.23 ± 1.18% and 53.77 ± 0.995% more effectively than D-OS (52.09 ± 0.222% and 49.72 ± 0.952%) in A549 and Caco-2 cells, respectively (Fig. 9). In a dose-dependent manner, A-OS exhibited stronger inhibitory effect on MMP2 and MMP9 with lower IC50 values (18.58% and 8.295%, respectively) than D-OS (23.61% and 13.16%, respectively) as shown in Fig. (10). Attractively, the polyunsaturated fatty acids (PUFAs) especially, ω-3 and ω-6 were recorded as selectively induced tumor cells apoptosis. The sensitivity of various cancer cells to different fatty acids were found to be variable depending on the type of cancer cell being tested and also the types and concentrations of the tested fatty acid [90]. Different research articles explained the positive effects of ω-6 fatty acids in controlling the human lung tumor cell growth in a concentration-dependent manner [91]. Furthermore, ω-3 PUFA triggers cancer cell apoptosis and synergizes to increase the sensitivity of tumor cells to conventional therapies, with interesting applications in cancers resistant to treatment [92]. According to Madhavi and Das [93], AA, GLA, DHA and EPA were found to be the greatest effective in inhibiting tumor cells growth comparing to ALA and LA that recorded lower effects even at higher concentrations. Kumar and Das [94] proved that linoleic acid at concentration of 40 µg/mL had the ability to inhibit cancer cells, whereas, the lower concentrations (5–10 µg/mL) enhanced the growth of nearly all types of cancer cells that were being tested in such study.

This tumoricidal action of fatty acids could be attributed to the increase in the generation of free radicals in the tumor cells [90]. Moreover, there is an evidence indicating that ω-6 PUFA and LA can be involved in both pro- and anti-cancer processes. For example increased the proliferation of the breast carcinoma cell line (BT-474 cell line) and the human lung cancer cell line (A549) in vitro, as well as promoting colon and prostate tumorigenesis and tumor growth in animal models [95, 96]. On the other hand, a high dose of LA could inhibits the proliferation of the colon cancer cell line (Caco-2) [97], while a high intake of LA can also show a protective effect against cancer development [98]. Based on this theory, different literatures proved the anticancer leverages of fatty acids, at least partly owing to their proapoptotic effects, especially against colorectal cancer (e.g., Caco-2, HT-29, HCT116, LoVo, SW480 and SW620 cell lines) [99], MHCC97L hepatocellular carcinoma cell line [100] and LNCaP, DU145 and PC-3 prostate cancer cell lines [101]. Furthermore, dihomo-γ-linolenic acid proved selective cytotoxic effects against A549 lung cancer cell line without affecting the normal cells [102]. Remarkably, the activation of caspases is the primary indicator of apoptosis-mediated cancer cell death. Caspase 3 is one particular caspase whose activation causes irreversible cell death. Previous studies illustrated that hen egg yolk PUFAs and Fish oil-enriched PUFAs activated caspase 3 in the treated melanoma cells and breast cancer, respectively [10, 103]. Moreover, both MMP2 and MMP9 are considered the main contributors in mediating invasion and metastasis of lung and colon cancers [104, 105]. PUFA (ω-3 and ω-6 fatty acids) revealed high inhibition potency in suppressing both MMP2 and MMP9 activities [11, 13, 29].

Finally, the former results of anticancer and antibiofilm proficiency of SCOs could be symbolized as prospective contenders for prophylactic programs by the dint of their environmentally-sound and biocompatibility. Besides, their unspecific mode of action triggers the appearance of mutant phenotypes with FAs-resistance is less problematic than commercial drugs. In this avenue, SCOs of the present study deemed as promising alternative anti-infective agents in various biotechnological applications like agriculture, medicine, nutraceuticals, feed additives and the cosmetic formulations. Presently, studies are profoundly under way to examine the efficacy of our SCOs in combinatorial treatment or drug amalgamation strategy with metal/ polymer nanomaterials, in lieu of using single agent. Such nanoformulations composites would be employed as an alternative wound dressing material in vitro study. Also, their exploitation as an anti-acne agent in skin ointments or gels is also run in parallel. However, in the avenue of food technology synchronizing with exploiting higher nutritional value of SCOs, which are rich in ω-3 and ω-6, oral tablets of SCOs and probiotics would be designed for promoting indigenous microbiota to facilitate the digestion process in human and livestock.

## Conclusion

This study highlighted the potency of the two oleaginous fungi (*Alternaria* sp. and *Drechslera* sp.), as promising factories, for their remarkable capability in economic production of ecologically friendly SCOs based on whey, as the main nutritive substrate. Fatty acid profile of both fungi revealed the presence of appreciated unsaturated fatty acids, which displayed superior anti-biofilm and anticancer activity. The overall data demonstrated antibiofilm potentiality of *Alternaria* oil sample in defeating all examined types of biofilm-forming pathogens comparing to *Drechslera* oil sample. On the other hand, data revealed that *Drechslera* oil sample is the most potent anticancer treatment against both A549 and CaCo-2 cell lines. Thus, the data gained from this study opens new perspectives in the field of biotechnology and considers superlative solution for the coming years to support cancer therapy and biofilm causing infections.

## Data Availability

The datasets analyzed during the current study are available in https://www.ncbi.nlm.nih.gov/nuccore/MH348917.1/ with accession number (MH348917.1) and https://www.ncbi.nlm.nih.gov/nuccore/MG582185.1/ with accession number (MG582185.1).

## Abbreviations

SCOs: Single cell oil
A-OS: *Alternaria *oil sample
D-OS: *Drechslera *oil sample
FTIR: Fourier transform infra-Red
Wi-38: Human normal fibroblast lung cells
A549: Human Lung adenocarcinoma, epithelial cells
CaCo-2: Human Colorectal adenocarcinoma, epithelial cells
IC50: The inhibition concentration 50
SI: Selectivityindex
FAs: Fatty acids
SFAs: Saturated fatty acids
MUFAs: Mono unsaturated fatty acids
PUFAs: Poly unsaturated fatty acids
ɷ-6: Omega 6
ɷ-3: Omega 3
*C. albicans*: *Candida albicans*
*S. aureuse*: *Stapylococcus aureuse*
*P. aeruginosa*: *Pseudomonas aeruginosa*
